# Groundwater Isolation Governs Chemistry and Microbial Community Structure along Hydrologic Flowpaths

**DOI:** 10.3389/fmicb.2015.01457

**Published:** 2015-12-22

**Authors:** Sarah Ben Maamar, Luc Aquilina, Achim Quaiser, Hélène Pauwels, Sophie Michon-Coudouel, Virginie Vergnaud-Ayraud, Thierry Labasque, Clément Roques, Benjamin W. Abbott, Alexis Dufresne

**Affiliations:** ^1^OSUR-UMR 6553 ECOBIO, Université de Rennes 1 and Centre National de la Recherche ScientifiqueRennes, France; ^2^OSUR-UMR 6118 Géosciences, Université de Rennes 1 and Centre National de la Recherche ScientifiqueRennes, France; ^3^BRGM, Laboratory DepartmentOrléans, France; ^4^OSUR-UMS 3343, Université de Rennes 1 and Centre National de la Recherche ScientifiqueRennes, France

**Keywords:** aquifers, hydrology, 16S rRNA, pyrosequencing, groundwater, bacterial communities, iron oxidation, nitrate

## Abstract

This study deals with the effects of hydrodynamic functioning of hard-rock aquifers on microbial communities. In hard-rock aquifers, the heterogeneous hydrologic circulation strongly constrains groundwater residence time, hydrochemistry, and nutrient supply. Here, residence time and a wide range of environmental factors were used to test the influence of groundwater circulation on active microbial community composition, assessed by high throughput sequencing of 16S rRNA. Groundwater of different ages was sampled along hydrogeologic paths or loops, in three contrasting hard-rock aquifers in Brittany (France). Microbial community composition was driven by groundwater residence time and hydrogeologic loop position. In recent groundwater, in the upper section of the aquifers or in their recharge zone, surface water inputs caused high nitrate concentration and the predominance of putative denitrifiers. Although denitrification does not seem to fully decrease nitrate concentrations due to low dissolved organic carbon concentrations, nitrate input has a major effect on microbial communities. The occurrence of taxa possibly associated with the application of organic fertilizers was also noticed. In ancient isolated groundwater, an ecosystem based on Fe(II)/Fe(III) and S/SO_4_ redox cycling was observed down to several 100 of meters below the surface. In this depth section, microbial communities were dominated by iron oxidizing bacteria belonging to Gallionellaceae. The latter were associated to old groundwater with high Fe concentrations mixed to a small but not null percentage of recent groundwater inducing oxygen concentrations below 2.5 mg/L. These two types of microbial community were observed in the three sites, independently of site geology and aquifer geometry, indicating hydrogeologic circulation exercises a major control on microbial communities.

## Introduction

Aquifers are populated by a vast diversity of microorganisms, some of which can play a critical role in subsurface biogeochemical cycling and pollutant degradation (Yagi et al., 2010). Because groundwater usually contains low concentrations of organic carbon, microbial life there depends mostly on oxidation and reduction of inorganic compounds for energy. Thus, geochemical conditions, and in particular the availability of electron donors and acceptors, are a major driver of microbial community composition and diversity in groundwater and the geological substratum (Akob et al., 2007; Boyd et al., 2007; Flynn et al., 2012, 2013; Nyyssönen et al., 2014).

Groundwater geochemistry is regulated in part by the distribution of groundwater flow paths and groundwater residence time (Tarits et al., 2006; Roques et al., 2014a). To date very few studies have addressed the effect of hydrology on groundwater microbial communities, and those that have were restricted to a single aquifer (Roudnew et al., 2010; Bougon et al., 2012; Larned, 2012; Lin et al., 2012; Zhou et al., 2012). It is therefore imperative to assess the effects of groundwater circulation on microbial communities. The comparison of several aquifers with well-defined hydrogeologic conditions allows to constrain the influence of groundwater flow in order to better understand biogeochemical functioning of groundwater ecosystems.

**FIGURE 1:**
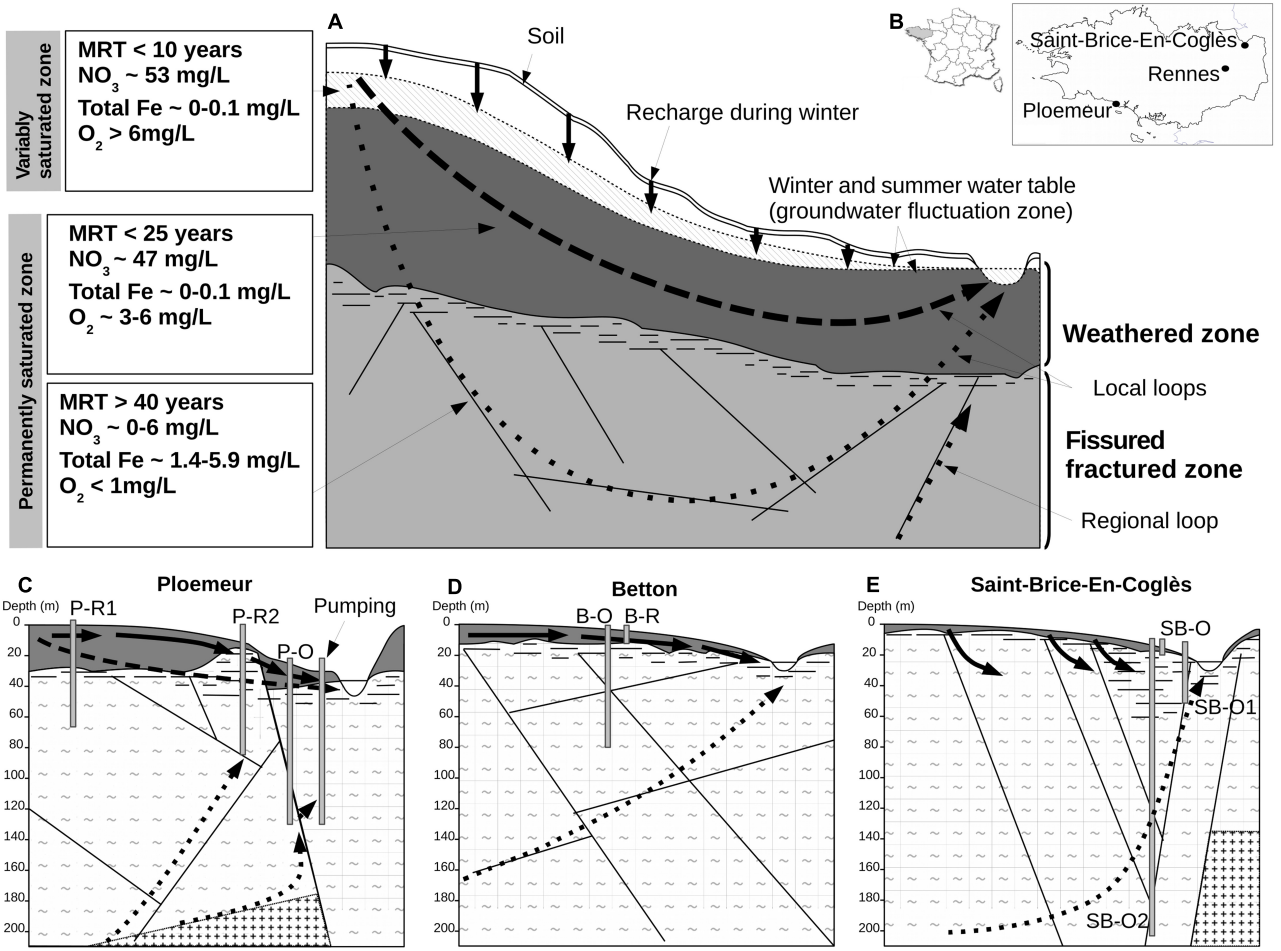
**Conceptual model of superficial and deep hydrogeologic flow paths or loops in a hard-rock aquifer and field-site characteristics.** In **(A)**, groundwater fluxes are mainly constrained by topographic relief, which creates hydraulic-pressure gradients. Transfers of O_2_- and NO_3_-rich recharge through the variably saturated zone of the weathered zone (solid vertical arrows) induce annual fluctuation of water table height. In the weathered zone, relatively short loops (represented by bold dashed arrows) have rapid water renewal resulting in short residence times, high O_2_ and NO_3_^-^ concentrations, and low concentrations of total dissolved iron. Longer hydrologic loops (represented by dotted arrows) pass through the fissured fractured zone of the bedrock. Groundwater along these loops has much longer residence times with very low rates of renewal (a few percent annually) inducing hypoxic conditions, very low NO_3_^-^ concentration, extended water-rock interaction, and elevated iron concentration. Part of a deep regional loop representing inter-watershed water transfer is depicted in panel **(A)**. In **(B)**, geographic location of the field-sites in the Brittany region is depicted. **(C–E)** show the main characteristics of each field-site: aquifer geometry, lithology, and configuration of wells and hydrogeologic loops. Lithology is represented by tilde signs (∼) for schist or plus signs (+) for granite. Abbreviations: MRT, mean residence-time of groundwater; P-R1, Ploemeur-Recent 1; P-R2, Ploemeur-Recent 2; P-O, Ploemeur-Old; B-O, Betton-Old; B-R, Betton-Recent; SB-O2, Saint-Brice-Old 2; SB-O, Saint-Brice-Old; SB-O1, Saint-Brice-Old 1.

Crystalline rocks represent about 35% of the continental surface (Blatt and Jones, 1975; Amiotte Suchet et al., 2003). They include crystalline aquifers also called hard-rock aquifers which constitute sensitive water resources that are increasingly exploited (Foster and Chilton, 2003; Mall et al., 2006). Water circulation in any aquifer is caused by the difference between the groundwater-table level in recharge zones high in the catchment, and the groundwater-table level in discharge zones in lowlands when the underground hydrogeologic catchment is similar to the hydrologic surface catchment. That difference induces a hydrogeologic gradient, generating hydrogeologic flow paths or loops along the catchment slope (see **Figure 1A** for a model of hydrogeologic circulations). More specifically, in hard-rock aquifers, the groundwater flow constitutes a topographic flow system mainly in the near-surface weathered zone, which constitutes the upper layer of these aquifers (Wyns et al., 2004; Ayraud et al., 2008). Hydrogeologic loops may extend beneath the weathered layer into the fractured compartment where circulation takes place exclusively in networks of fractures (Wyns et al., 2004; Ayraud et al., 2008). Large-scale regional loops may connect recharge and discharge zones over larger distances than the catchment size (i.e., hydrogeologic catchment is larger than hydrologic catchment), causing inter-watershed exchanges and making it more difficult to define the recharge zone supplying large fracture discharges (Roques et al., 2014a; Aquilina et al., 2015). There is a partitioning between the weathered formation and the deeper fractured rock formation which have different permeability and porosity resulting in contrasting mean residence times (Ayraud et al., 2008). This partitioning results in short mean residence times in the superficial layer (<25 years) and long residence times (ranging from 40 years to millennia) in the deeper fractured formation which is thus considered as partly isolated as compared to the surface layer which is actively recharged.

Differences in hydrogeologic circulation and residence time result in distinct groundwater geochemical composition, e.g., modulating contributions of atmospheric oxygen and agricultural eﬄuents. Annual recharge provides a relatively constant supply of oxygen from the surface to the weathered zone of hard-rock aquifers, as well as chemical elements resulting from intensive agriculture such as nitrate and leaching of fertilizers. Conversely, groundwater in the fractured zone is relatively protected and relatively isolated (Ayraud et al., 2008). However, even regional hydrogeological loops are mixed to a certain degree to modern groundwater from the weathered zone and do not represent fully isolated ecosystems.

In this study, we assessed how groundwater partitioning and isolation drives both groundwater hydrogeochemistry and active microbial community structure in hard-rock aquifers by comparing surface recent groundwater considered as an opened ecosystem, and old deep groundwater considered as partly isolated, i.e., with low surface groundwater renewal.

High-throughput sequencing based approaches such as 16S rRNA gene profiling and metagenomics have made it possible to explore complex groundwater microbial community and metabolism (Hemme et al., 2010; Lin et al., 2012; Smith et al., 2012; Wrighton et al., 2012; Castelle et al., 2013; Nyyssönen et al., 2014; Brown et al., 2015). Here, we collected groundwater samples from three geographically distinct aquifers (Brittany, France, see **Figure 1**) where hydrogeologic and hydrogeochemical parameters have been monitored for the last 5 years allowing to determine residence times of groundwater. We used pyrosequencing of 16S Ribosomal RNA amplicons to characterize groundwater microbial communities, and to infer putative microbial processes. As dissolved organic carbon concentrations are below 1.3 mg/L in all the investigated sites (Ayraud et al., 2006; Aquilina et al., 2015; Ploemeur monitoring database http://hplus.ore.fr/), we hypothesized that heterotrophy remains limited at all depths. We also hypothesized that nitrate and oxygen are the major drivers in the weathered zone and that chemolithotrophic metabolisms linked to iron and reduced sufur oxidation or sulfate reduction may occur in the fractured zone which represents an interesting situation of very slow nutrient renewal, i.e., a transition state close to full isolation. The investigation of three aquifers at the regional scale, with different hydrological conditions which are well constrained, allowed us investigating a more general case to determine the relationships between microbial diversity and hydrology.

## Materials and Methods

### Hydrogeologic Context

Hard-rock aquifers from a geologic and hydrogeologic point of view typically consist of two layers (a surficial weathered layer and a deep fractured layer) with a sharp boundary separating two distinct hydrogeologic regimes resulting in contrasting hydrogeological properties : a topographic flow regime and deeper local or regional groundwater loops (**Figure 1A**) (Wyns et al., 2004; Ayraud et al., 2008). However, the geochemical composition of groundwater bodies does not always strictly reflect the surface/deep fractured compartmentalization as it may be influenced by regional hydrogeologic loops crossing watershed boundaries (**Figure 1A**). In this study, we have collected samples at various positions along hydrogeologic loops, determined by residence time, in order to access recent and old groundwater in different geologic contexts (**Figures 1C–E**). Three hard-rock aquifers in Brittany (Western France) were investigated near the cities of Ploemeur (N47.735°, W-3.427°), Betton (N48.182°, W-1.643°) and Saint-Brice-En-Coglès (N48.411°, W-1.365°) (**Figure 1B**). Field-site and well attributes are represented in **Figures 1C–E** of. Wells are coded with the first letter of the field-site (P for Ploemeur, B for Betton, or SB for Saint-Brice-En-Coglès) followed by the mean groundwater residence time (O for old i.e., >40 years, or R for recent, i.e., <25 years). Mean groundwater residence time was determined by the analysis of dissolved anthropogenic gasses (see Ayraud et al., 2008) for further details about the groundwater dating protocols and further references herein). The Ploemeur site is an active recharge zone occurring on a geologic boundary between a Hercynian granite and a schist bedrock, and is influenced by intensive pumping (1 million m^3^ annually) (Le Borgne et al., 2004; Jimenez-Martinez et al., 2014) (**Figure 1C**). This site includes: a deep well with old groundwater (P-O) sampling the discharge portion of a regional hydrogeologic loop; the P-R1 well that samples shorter circulation loops with recent recharge groundwater; and the P-R2 well, which is a mix of old and recent groundwater related to the active pumping at the Ploemeur site (Tarits et al., 2006; Ayraud et al., 2008). The Betton site is underlain by Proterozoic schists (**Figure 1D**). The two wells sampled in Betton intersect very distinct groundwater components. The B-R well is close to the surface and is fed by short circulation loops with short groundwater residence times, while the B-O well reaches a deep groundwater compartment with a residence time of several 1000 years (Aquilina et al., 2015; Armandine Les Landes et al., 2015). The St-Brice site is located on a metamorphic formation named the Hornfel schists, close to a Cadomian granitic formation (**Figure 1E**). It is at the discharge zone of a regional groundwater loop. Groundwater flows along a large fault zone (Roques et al., 2014b) and is characterized by a mean residence time of several 100 years (Roques et al., 2014a). SB-O2 and SB-O1 wells are fed by this groundwater flow, though they were sampled at distinct depths (216 and 80 m), while the SB-O well is fed by more superficial loops resulting in a natural mixing of old and recent groundwater. All investigated wells have a screen length ranging from 1 to 2 m.

The upper part and recharge zone (i.e., topographic flow system) of hard-rock aquifers in Brittany has been investigated; especially for determining nitrate pollution transfer in groundwater (Legout et al., 2005, 2007; Martin et al., 2006; Aquilina et al., 2012; Roques et al., 2014b). Both modeling and groundwater residence time analysis show that groundwater mean vertical velocity is about a few meter per year (Legout et al., 2005; Ayraud et al., 2008). These studies have shown that recharge processes induce rapid transfer through the unsaturated zone which is only a few meter thick and intensive vertical mixing (Rouxel et al., 2011; Bougon et al., 2012). It can thus be assumed that Brittany aquifers present intensive renewal of nitrate and oxygen nutrients in the weathered and/or recharge section. Short residence times are associated to these hydrologic conditions as, except in the variably saturated zone, groundwater presents residence time in the range 5–10 years in the upper meter of the permanently saturated zone.

In the fractured section of these aquifers, groundwater mean vertical velocity is close to 1 m per year (Ayraud et al., 2008). However, such velocity is more difficult to interpret as groundwater flow path strongly depends on the structure of the fracture network. Within the three aquifers investigated, old groundwater represent regional loops with high residence time. As some analysis (^14^C, ^36^Cl) indicate large residence times (Roques et al., 2014a; Aquilina et al., 2015), the CFC residence times represent a mixing degree between much older groundwater and recent one. We thus consider that residence times used in this study can be used as a proxy of the mixing degree of these groundwater with recent surface groundwater, i.e., the low degree of oxygen and nitrate potential nutrients to the deep ecosystem. High residence time characterize concentrations that are always very low and vary with time although they are usually not completely absent. As the mixing degree is quite low, we consider that deep groundwater can be considered as isolated as compared to surface recent groundwater.

### Groundwater Sampling and Hydrochemical Analyses

Groundwater samples were collected between October and November 2012. All wells were analyzed for chemistry and all but P-O and SB-O were analyzed for microbial community composition. Sampling was done using a Grundfoss MP1 pump (flow rate : 150–200 L/hr), after purging the tubing and waiting for stabilization of conductivity, pH, redox potential, temperature and oxygen measurements (typically occurring within 30 min). Temperature, conductivity, and oxygen were measured with a WTW315i-CondOx probe, pH with a Combined SenTix 50 electrode, and redox potential with a Pt Ag/AgCl electrode (Mettler Pt 4805). For chemical analyses, approximately 50 ml of groundwater was filtered to 0.22 μm into polytetrafluoroethylene (PTFE) acid-rinsed bottles. Cations were measured with an ICP-MS HP 4500, anions were analyzed by ion chromatography (Dionex DX-100), and carbon was analyzed with a Shimadzu TOC 5050A Analyzer at the Chemistry facility of the Observatoire des Sciences de l’Univers de Rennes (OSUR, University of Rennes 1 – CNRS). We used a principal component analysis (PCA) to assess the variability of hydrochemical parameters over a time period of 5 years, including samples from the two groundwater bodies not included in the high-throughput sequencing (P-O and SB-O). Hydrochemical measurements carried out prior 2012 were performed using the same protocol as described in this paper. The hydrochemical data are available at the website of the H^+^ network of hydrogeological research (hplus.ore.fr). PCA was performed using hydrogeochemical parameters with correlation coefficients calculated using R and lower than 0.8 (Supplementary Table S1).

### RNA Extraction, 16S rRNA RT-PCR and Construction of 16S rRNA 454 Libraries

For each groundwater well, triplicate 5 l samples were filtered to 0.22 μm with polyvinylidene fluoride filters (Durapore). RNA extractions were performed with a protocol optimized to improve the removal of organic contaminants (Quaiser et al., 2014). The lysis buffer contained beside CTAB and guanidium thiocyanate, polyvinylpyrrolidone that retains selectively organic compounds as humic acids and fulvic acids (Quaiser et al., 2002). The complete removal of DNA was confirmed by PCR using 16S rRNA gene primers (U27F-U1492R). The 16S rRNA RT-PCR was performed according to manufacturer’s instructions (Roche, Titan One RT-PCR kit) using primers 515F (5′-GTG CCA GCM GCC GCG GTA ATA C-3′) and 926R (5′-CCG TCA ATT CCT TTG AGT TT-3′), targeting the V4 and V5 hypervariable regions of Bacteria, and fused to Roche sequencing adaptors and Multiplex IDentifiers (MID). With each RNA extract, two independent RT-PCRs were performed with fusion primers containing distinct MIDs. Amplicons from each RT-PCR were quantified and multiplexed. Library preparation, emulsion PCR, and pyrosequencing on a Roche/454 GS FLX Titanium instrument were performed according to manufacturer’s protocol (454 Life Sciences/Roche Applied Biosystems, Branford, CT, USA) at the Environmental Genomics facility at OSUR. These sequence data have been submitted to the ENA databases under the accession No. PRJEB8879.

### Community Sequence Analyses

Sequence analyses were performed using Mothur (v1.31.0, Schloss et al., 2009) and DNACLUST (Ghodsi et al., 2011). Raw reads were filtered to remove those with (i) homopolymers longer than six nucleotides, (ii) undetermined nucleotides, (iii) anomalous length, (iv) one difference, or more in the primer and barcode sequence. Reads were also trimmed based on quality score associated to each nucleotide. Quality filtered reads were then clustered into operational taxonomic units (OTU) with a 97% identity threshold.

We used the distribution of sequences in samples and replicates of amplification to remove spurious OTU that could inflate diversity estimates. Only OTUs containing at least two 100% identical sequences, appeared independently in different replicates were retained for the subsequent analyses. Chimeric sequences were identified with the chimera.uchime command of Mothur and eliminated from the dataset. Valid sequences in OTUs were compared to the SSU rRNA Silva database (Quast et al., 2013, version 115 of August 23, 2013) to determine taxonomic affiliations. We computed richness (*Sobs*) and rarefaction curves as well as diversity (Simpson’s index) with Mothur. The community structures were compared with non-metric multidimensional scaling (NMDS) after calculating a Bray–Curtis dissimilarity matrix with vegan (R-package vegan, Oksanen et al., 2015). The fitting of environmental variables to the ordination plot was performed with vegan and the significance was obtained with a 1000 permutations test. Tested parameters were NO_3_^-^, SO_4_^2-^, O_2_, Cl^-^, Na^+^, Mn_(total)_, Fe_(total)_, Br^-^, Mg^2+^, K^+^, Ca^2+^, Cu^2+^, Zn^2+^, PO_4_^2-^, and total organic carbon concentrations, temperature, conductivity, redox potential, pH, groundwater residence time, and depth. To address more directly how environmental parameters and individual OTUs were related, we performed a regularized Canonical Correlation Analysis (rCCA) between relative frequencies of OTUs and environmental parameters with the R package FRCC (Cruz-Cano and Lee, 2014). We visualized results of the rCCA analysis using the approach developed by Patel et al. (2010) A Network was built from the rCCA results. Each node represents either an OTU or an environmental parameter. Edges connecting nodes are weighted by calculating the dot product between the structural correlations of the first and fifth dimensions of the rCCA (Patel et al., 2010). Only edges with value weights above 0.3 were used to build the network. Network visualization were performed with Cytoscape (Shannon et al., 2003). Additional information were mapped on each node to facilitate the interpretation of the network. This information included the taxonomic assignment and the maximal abundance of each OTU, and the sample where each OTU is the most prevalent. Analysis of the network topology allowed identifying the sets OTUs and environmental features that are the most correlated to each other.

## Results

### Geochemical Characterization of Groundwater

**FIGURE 2:**
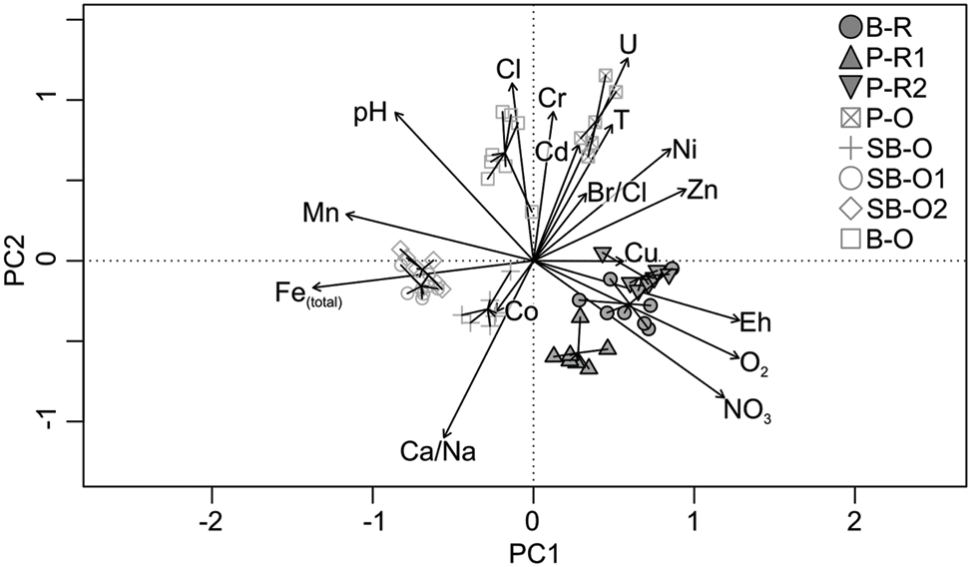
**Principal component analysis (PCA) plot of hydrogeochemical data measured in groundwater from three hard-rock aquifers.** The first and second principal components (PC1 and PC2) account for 27 and 17% of variance, respectively. The plot includes groundwater samples from all wells, including P-O and SB-O which were not used in the microbial diversity analysis. Recent groundwater samples are represented by closed symbols and old groundwater samples by open symbols. Abbreviations : B-R, Betton-Recent; P-R1, Ploemeur-Recent 1; P-R2, Ploemeur-Recent 2; P-O, Ploemeur-Old; SB-O, Saint-Brice-Old; SB-O1, Saint-Brice-Old 1; SB-O2, Saint-Brice-Old 2; B-O, Betton-Old; T, temperature.

The hydrochemical variations between different groundwater bodies were visualized by PCA (**Figure 2**). The first PCA axis separated recent groundwater samples (B-R, P-R1, and P-R2) with an average residence time of 16 years, from old groundwater samples (SB-O2, SB-O1, SB-O, P-O, and B-O) with a residence time greater than 40 years (Supplementary Table S2). Recent groundwater bodies had variable but always high nitrate (47–53 mg/L) and oxygen concentrations (O_2_ : 6–10 mg/L), and high redox potentials (>250 mV) (Supplementary Table S2). Conversely, old groundwater samples exhibited high iron (>1 mg/L), manganese, and inorganic carbon (IC) concentrations as well as higher pH and more anoxic conditions (Supplementary Table S2). Old groundwater bodies had nitrate and oxygen concentrations that varied with time but ranged from below detection limit to 1.5 and 2.5 mg/L, respectively. Although samples in the PCA represent 5 years of monitoring covering diverse hydrologic conditions (periods of recharge and discharge and various levels of precipitation) the distinction between recent and old groundwater appears to be the major control on water chemistry. This is particularly clear for the recent groundwater bodies, which group closely despite representing three distinct geological sites. Higher variability between the old groundwater bodies is apparent in the PCA where B-O and P-O were associated with higher chloride, sulfate and sodium concentrations. Most saline groundwater bodies (B-O and P-O) were characterized by higher sulfate concentrations as chloride concentration was strongly correlated to sulfate concentration (not shown on the PCA plot, *R*^2^ = 0.971). The greater salinity of some groundwater bodies is due to complex site history described elsewhere (Aquilina et al., 2015; Armandine Les Landes et al., 2015).

### Groundwater Microbial Communities

**FIGURE 3:**
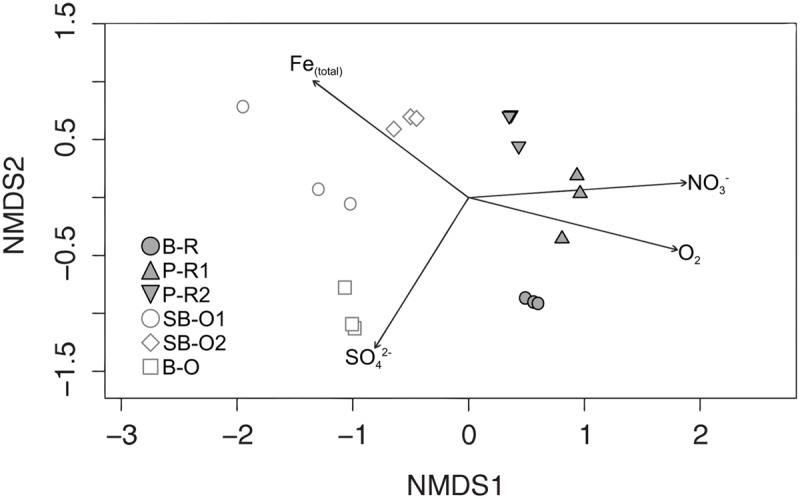
**Non-metric multidimensional scaling (NMDS) plot of Bray–Curtis dissimilarities between groundwater communities and vector fitting of hydrogeochemical variables to community ordination.** Arrows represent fitted hydrogeochemical variables with *p* ← 0.001. Recent groundwater samples are represented by closed symbols and old groundwater samples by open symbols. Abbreviations: B-R, Betton-Recent; P-R1, Ploemeur-Recent 1; P-R2, Ploemeur-Recent 2; SB-O1, Saint-Brice-Old 1; SB-O2, Saint-Brice-Old 2; B-O, Betton-Old; Fe_(total)_, total disssolved iron.

**FIGURE 4:**
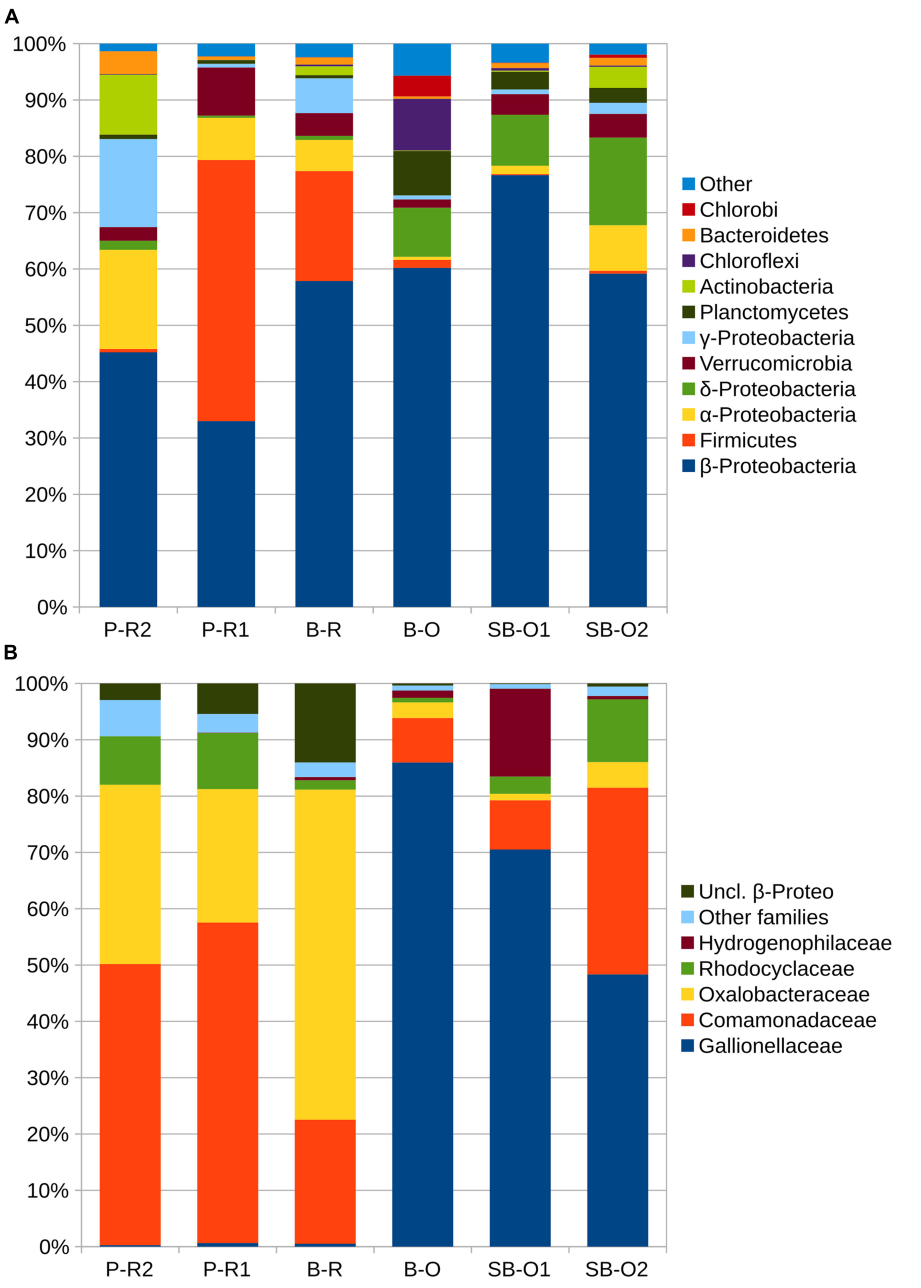
**Taxonomic composition of groundwater samples.** In **(A)** percentages of sequences assigned to the main phyla of bacteria. For sequences assigned to *Proteobacteria*, percentages represent the class level. In **(B)** is presented percentages of sequences for families of *Betaproteobacteria*. Taxa with less than 10 sequences detected are gathered in the category ≪ other ≫ in **(A)** and ≪ other families ≫ **(B)**. Abbreviations: P-R2, Ploemeur-Recent 2; P-R1, Ploemeur-Recent 1; B-R, Betton-Recent; B-O, Betton-Old; SB-O1, Saint-Brice-Old 1; SB-O2, Saint-Brice-Old 2; uncl., unclassified.

A total of 3910 OTUs were identified from half a million quality-filtered sequences from all groundwater samples. Individual wells ranged from 611 OTUs to 1827 OTUs (P-R1 and B-R, respectively). Computation of OTU richness and Simpson diversity index did not allow the separation of microbial communities according to their geographical location or sampling depth (Supplementary Figure S1). The dissimilarity in microbial community structure between samples was visualized on an NMDS plot (**Figure 3**). As in the PCA plot based on groundwater chemistry, there was a clear distinction between old (SB-O2, SB-O1, and B-O) and recent groundwater samples (P-R2, B-R, P-R1). The environmental parameters fitting revealed that differences in community structure were significantly correlated with a small number of environmental parameters: Fe_(total)_, SO_4_^2^**^-^**, NO_3_**^-^**, and O_2_ concentrations (*p* < 0.001). In total, fifteen bacterial phyla were identified in the groundwater sequences (**Figure 4A**). For old groundwater communities, the majority of sequences were assigned to Betaproteobacteria (accounting for 59–77% of sequences) followed by Deltaproteobacteria (8–16%) and Planctomycetes (3–8%). Sequences of Chlorobi and Chloroflexi accounted for 9% of the sequences in B-O whereas Verrucomicrobia represented a significant portion of sequences in SB-O1 and SB-O2 (3 and 4%, respectively). Taxonomic profiles show that Betaproteobacteria also accounted for a large proportion of sequences in recent groundwater, with 58% in B-R, 45% in P-R2, and 33% in P-R1. However, we found that Firmicutes represent another dominant phylum in recent groundwater communities with 20 and 46% of the sequences ascribed to this phylum in B-R and P-R1, respectively. The majority of Firmicutes sequences were assigned to the *Clostridium* genus (Supplementary Figure S2). Despite their strong similarity in groundwater chemistry, P-R1 and P-R2 samples had distinct microbial communities, with P-R2 showing more numerous sequences affiliated to *Alpha-* (18%), Gammaproteobacteria (16%), and Actinobacteria (11%) whereas communities from P-R1 were dominated by Firmicutes (46%).

**FIGURE 5:**
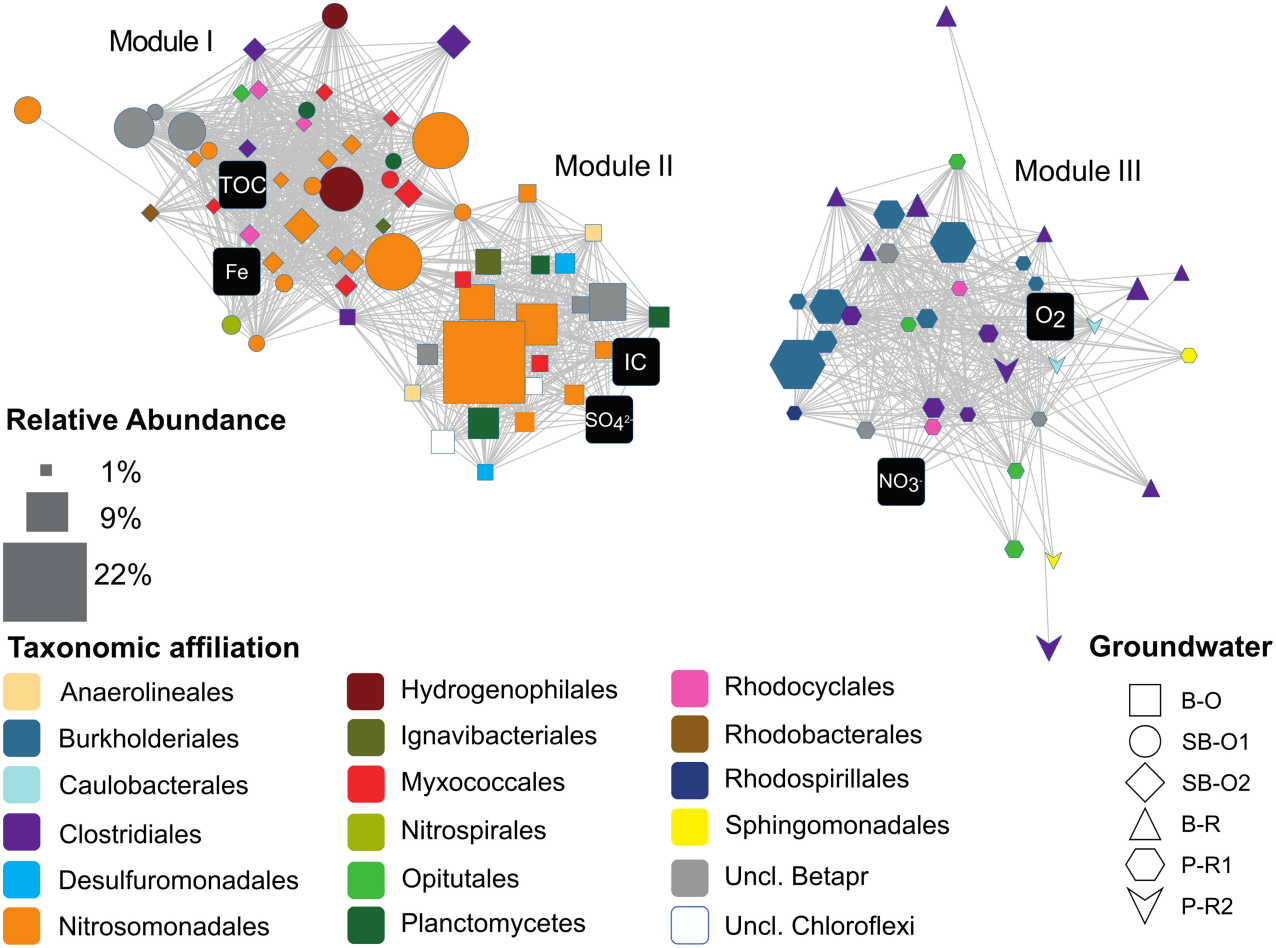
**Network of structural correlations between and among OTU and hydrogeochemical variables.** Edges represent positive and significant correlations. Nodes depicted as black squares represent hydrogeochemical variables. The other nodes are OTUs with relative abundances ≥1%. Additional information is mapped onto OTU nodes to facilitate interpretation of the network: node size is the maximum abundance of the OTU across samples; node shape represent samples where the OTU is the most prevalent; node color represents the taxonomic assignment. Abbreviations: TOC, Total Organic Carbon; IC, Inorganic Carbon; Fe, total dissolved iron; Uncl, unclassified; Betapr, Betaproteobacteria; B-O, Betton-Old; SB-O1, Saint-Brice-Old 1; SB-O2, Saint-Brice-Old 2; B-R, Betton-Recent; P-R1, Ploemeur-Recent 1; P-R2, Ploemeur-Recent 2.

The distinction in community composition between old and recent groundwater samples was more clear at the family level within Betaproteobacteria (**Figure 4B**). In old groundwater, the majority of sequences were attributed to Gallionellaceae, accounting for 25 to 44% of sequences. In SB-O1, a substantial proportion of sequences was assigned to the Hydrogenophilaceae (9%). Both families were rarely detected in recent groundwater samples (<0.3%). In recent groundwater samples, 26–57% of Betaproteobacteria sequences were assigned to Comamonadaceae (12–20%), Oxalobacteraceae (7–33%), and Rhodocyclaceae (1–4%). Conversely, these three families only accounted for 8–25% in old groundwater. NMDS allows the comparison of communities on a dissimilarity matrix basis. To further investigate the interactions between environmental parameters and specific microbial community members, we performed a rCCA. Positive significant correlations between and among OTUs and hydrogeochemical parameters were visualized through a network with three modules (or sub-networks), each corresponding to a different hydrogeochemical context (**Figure 5**). Dominant OTUs in old groundwater form two highly interconnected modules (modules I and II). However, a clear separation by site can be observed as OTUs primarily from SB-O1 and SB-O2 (Saint-Brice-En-Coglès) form a distinct module from OTUs primarily detected in B-O (Betton). Both modules I and II were dominated by OTUs of Nitrosomonadales (mainly Gallionellaceae), Planctomycetes (Phycisphaerae, *OM190*, and Planctomycetacia class) and Myxococcales. Module I also contained two large OTUs of Hydrogenophilales (mainly *Sulfuricella*) and many OTUs of Rhodocyclales and Clostridiales. All the OTUs in this module were associated to the total Fe concentration. Module II also gathers OTUs of Ignavibacteriales and Desulfuromonadales and is characterized by the association of OTUs with SO_4_^2-^ and IC concentrations. Module III corresponds to a group of OTUs mainly detected in recent groundwater and associated with NO_3_**^-^** and O_2_ concentrations. This module includes OTUs of Clostridiales (mainly *Clostridium*), Burkholderiales (mainly Comamonadacaeae and Oxalobacteraceae), Caulobacterales, Opitutales (mainly *Opitutus* genus), Rhodocyclales (mainly *Propionivibrio* genus), and Sphingomonadales (*Sphingobium* genus). This module showed that sampling site had no effect, as OTUs detected in samples from Ploemeur (P-R1 and P-R2) and Betton (B-R) are all connected together.

## Discussion

### Denitrification Processes in the Superficial Ecosystem

Microorganisms detected in recent groundwater were highly associated with NO_3_^-^ and O_2_ concentrations (**Figures 3** and **5**). Indeed, recent groundwater bodies had very high NO_3_^-^ concentrations (from 47 to 53 mg/L) due to widespread application of organic and inorganic fertilizers on agricultural fields since the 1980s (Aquilina et al., 2012). Recent groundwater samples had a large portion of sequences affiliated with *Comamonadaceae* and *Oxalobacteraceae*. Both of these families contain many genera of potential denitrifying bacteria such as *Simplicispira, Rhizobacter, Curvibacter, Aquabacterium, Polaromonas* (Comamonadaceae, Betaproteobacteria), and *Rugamonas* (Oxalobacteraceae, Betaproteobacteria). The presence of these bacteria in conjunction with high NO_3_^-^ concentration suggests that persistent inputs of NO_3_-rich water could favor the development of a guild of denitrifying bacteria, representing a profound change in community structure and microbial processes in recent groundwater. However, despite the presence of putative denitrifying Betaproteobacteria, there is no clear evidence of substantial denitrification in these groundwater bodies. Low organic carbon concentrations in groundwater (ranging from 0.24 to 1.3 mg/L over the 5 years) can limit heterotrophic denitrification (Korom, 1992; Starr and Gillham, 1993; Rivett et al., 2008). Autotrophic denitrification linked to Fe oxidation can occur in groundwater (Smith et al., 1994; Rivett et al., 2008; Weymann et al., 2010) but is likely limited in this aquifer section by the low Fe concentration observed in recent groundwater samples.

These results are in good agreement with previous microbial analysis of a surficial granitic aquifer in Brittany (Bougon et al., 2012) which investigated the communities at a depth ranging from 6 m down to 15 m below the soil surface. This study identified a large proportion of nitrate reducing bacteria in the aquifer although no clear and complete denitrification processes could be detected. These results and the present study indicate that denitrification remains probably a key function in the ecosystem although it does not accounts for a complete nitrate consumption. The strong link between NO_3_^-^ concentration and the proportion of denitrifiers in our dataset suggests that perturbations of the nitrogen cycle in surface catchments can substantially impact microbial community structure in groundwater. Theses results suggest a limited denitrification capacity that could constitute a limit to groundwater remediation.

### Anthropogenic Influence in the Superficial Ecosystem

Although the mean residence time in recent groundwater is approximately 18 years (Ayraud et al., 2008), the high O_2_ concentration suggests inflows of oxygenated surface water through the recharge process. Geochemical tracers and groundwater dating methods showed regular inputs of surface precipitation and soil-water to the top of these topographic groundwater bodies (Legout et al., 2007; Aquilina et al., 2012). Therefore, an alternative explanation for the link between NO_3_^-^ concentration and proportion of potential denitrifiers may be direct transfer of microorganisms from the soil or underlying unsaturated zone where high organic carbon concentration could sustain denitrification. Indeed, nutrients and denitrifying activity are higher in the soil and near-surface unsaturated zone than in the deeper permanently saturated zone (Legout et al., 2005). Analysis of microbial diversity during one hydrological cycle at depth of 6 to 15 m below soil surface (Bougon et al., 2012) showed that microbial diversity at 15 m depth (below soil surface) was more homogeneous than at shallower 6 m depth. Close to the surface, rapid transfer during recharge processes (high groundwater levels) induced a modification of the community structure although diversity in the 6 m shallow groundwater remained similar to the 15 m depth ecosystem during low groundwater-level periods.

In addition to potentially denitrifying proteobacteria, recent groundwater from P-R1 and B-R had a high proportion of 16S rRNA transcripts affiliated to Firmicutes, many of which were assigned to the *Clostridium* genus (Supplementary Figure S2). This genus is often associated with mammalian gut habitats or soil (Lopetuso et al., 2013) and *Clostridium perfringens* has been proposed as an indicator of fecal pollution (Araujo et al., 2004; Mueller-Spitz et al., 2010). Contamination of groundwater by rumen-related bacteria including *Clostridium*-affiliated microorganisms was demonstrated in an intensive farming zone in Korea, from the comparison of livestock-contaminated wastewater and uncontaminated groundwater in sampling sites located 400m apart (Cho and Kim, 2000). Thus, we suggest that the strong prevalence of amplicons attributed to *Clostridium* combined with the high nitrate load in recent groundwater can also be attributed to the transport of bacteria from livestock waste during the recharge process. A similar result was also obtained by Bougon et al. (2012) with *Bacillus* identification.

### A Fe-Based Ecosystem in Isolated Groundwater

Old groundwater in all sites was characterized by high Fe concentrations. In sequences from old groundwater, Gallionellaceae sequences were dominant. This family is primarily composed of autotrophic Fe-oxidizers including *Gallionella* and *Sideroxydans* genera that grow optimally in neutral, microaerophilic, Fe-rich environments (Hallbeck and Pedersen, 1990; Emerson et al., 2013). Abundance of 16S rRNA amplicons does not allow direct quantification of microbial cells activity (Větrovský and Baldrian, 2013) but it does reflect *in situ* transcriptionally active cells, thereby avoiding bias due to dead or dying cells. It constitutes a powerful tool to identify microorganisms with fundamental activities for ecosystem functioning.

Our analysis of rRNA amplicons strongly suggests that Gallionellaceae are the main primary producers in old groundwater where their autotrophic activity could sustain the development of other heterotrophic microorganisms. Their inability to oxidize Fe when O_2_ concentration is high (Druschel et al., 2008) explains why Gallionellaceae are restricted to deeper isolated groundwater. However, the distribution of Gallionellaceae also suggests minimal renewal of old groundwater with oxygenated recent groundwater from surface, creating chemical conditions (neutral-pH, Fe-rich, O_2_-poor groundwater) favoring the development of a Gallionellaceae-dominated community. This interpretation corresponds with the geochemical data indicating that contribution from old groundwater bodies makes up a very small percentage of near-surface groundwater. Each year, the percentage of recent groundwater flushing into the old groundwater body is about 1 to 5% of the old groundwater body (Aquilina et al., 2015). The prevalence of *Gallionellaceae* in our dataset raises the question of the Fe origin in old groundwater. Some of the Fe(II) concentration may come from the dissolution of sulfur-bearing minerals (pyrite). Pyrite is very common in crystalline rocks. This mineral dissolution can occur either abiotically through chemical oxidation with O_2_, which must be negligible here because pH are close to neutrality_.,_ or can be biologically facilitated by NO_3_^-^ reducing microorganisms (Melton et al., 2014). Beside the release of reduced iron, microbially driven dissolution of pyrite leads to the production of sulfate in significant proportions. Analysis of SO_4_^2-^ concentrations and S-_SO4_ isotopic ratios indicate that pyrite dissolution occurred at the Ploemeur and St-Brice sites (Pauwels et al., 2010; Roques et al., 2014a). The relative importance of sulfide oxidation is difficult to determine, because SO_4_^2-^ concentrations are highly variable. In B-O, where SO_4_^2-^ concentrations are the highest, SO_4_^2-^ concentration may be the result of marine SO_4_^2-^ intrusion in the aquifer two million years ago (Aquilina et al., 2015; Armandine Les Landes et al., 2015).

However, in this old groundwater, SO_4_^2-^ to Cl^-^ ratios are higher than ratios found in seawater and precipitation, suggesting a supplementary SO_4_^2-^ source such as microbially mediated oxidation of sulfur-bearing minerals. The genome of *Sideroxydans* ES-1 includes several gene clusters involved in reduced sulfur oxidation. The ability of the ES-1 strain to grow using thiosulfate as the sole source of electrons supports the hypothesis that some members of the Gallionellaceae family can use reduced sulfur oxidation to produce energy (Emerson et al., 2013). However, to date, no evidence has been found that *Sideroxydans* ES-1 is able to couple iron or sulfide oxidation to nitrate reduction. Thus, further investigation is necessary to identify microorganisms actively involved in the weathering and dissolution of minerals and in the release of Fe and SO_4_^2-^ in crystalline aquifers. complex

In addition to sequences of bacteria involved in the oxidation of Fe and reduced sulfur compounds, all old groundwater samples contained sequences of microorganisms potentially involved in Fe or SO_4_^2-^ reduction. The Myxococcales (39–85% of the Deltaproteobacteria, Supplementary Figure S3) order shows sequences belonging to the *Anaeromyxobacter* genus reported as a Fe(III) reducer (Supplementary Figure S4; Sanford et al., 2002). The Desulfobacterales (1–31% of the Deltaproteobacteria) order includes several genera which have been described as SO_4_^2-^ reducers such as *Desulfobulbus, Desulfatirhabdium, and Desulfosalsimonas* (Supplementary Figures S5A,B; Balk et al., 2008; Kjeldsen et al., 2010; Pagani et al., 2011). Together, the simultaneous occurrence of Fe and reduced sulfur oxidizers and reducers in old groundwater hints at the existence of a complete microbial redox cycle for both elements. Due to the different proportions observed in the amplicon datasets, the oxidative portion of the cycle performed by Gallionellaceae appears to be predominant, thereby controlling the flow of carbon in old groundwater. Yet, we cannot rule out that iron-reducing bacteria might be underrepresented in our dataset because of a sampling bias, as they have the ability to thrive attached onto the aquifer substratum rather than suspended into groundwater (Flynn et al., 2008).

### Hydrogeologic Controls

Our results show that geochemistry and microbial community structure do not directly depend on sampling depth or location in the surficial weathered or deep fractured compartment of crystalline aquifers. Conversely, a clear distinction is observed between recent and old groundwater bodies, evident in both their geochemical and microbial signatures. Recent groundwater corresponds to the weathered part and the recharge zone of hard rock aquifers, i.e., to topographic flow systems. These groundwater are highly influenced by rapid transfer from the soil and unsaturated zone and present high oxygen and nitrate concentrations. Old groundwater correspond to deeper hydrogeologic loops where very high residence time groundwater slowly mixes with a low percentage of recent groundwater, resulting in very low but not null oxygen and nitrate concentrations. Thus, groundwater isolation along the hydrogeologic loops following active recharge zones, determine the fluxes of nutrients, energy and microorganisms circulating within the subsurface habitats.

From our analysis of recent groundwater, the massive input of NO_3_^-^ and the transport of microorganisms from the soil constitute major drivers for groundwater chemistry and microbial community composition that seem to overwhelm other factors such as lithology (granite or schist) or aquifer geometry (depth of the weathered zone). Thus, short or rapid groundwater circulation in topographic systems allow the connection between surficial habitats and groundwater bodies. This connection occurs mainly during the recharge period in winter while evapotranspiration is minimum and soil moisture is high, allowing vertical water transfers. This recharge process is not uniform and while it predominantly occurs as a slow transfer of soil water to the water table (about 1 m per year), rapid water transfer can occur via macropores such as dead root conduits that transport water from soil surface to the groundwater table in a matter of hours (Legout et al., 2007). Following hydrogeologic loops, the recharge water moves downward while microorganisms consume oxygen and nutrients. Within a catchment, except in discharge zones, one would expect oxygen, and nutrients to decrease while residence time increases with depth due to deepening hydrogeologic loops. However, this was not observed in the data where recent groundwater and old groundwater were clearly distinct. In the Ploemeur site, recent groundwater extends to the fractured layer because the fracture network is well connected to the recharge zones and the groundwater velocity is high. In the subsurface, further down the hydrogeologic loop, old groundwater geochemistry and microbial community structure appear to be tightly related to high Fe concentrations and Gallionellaceae prevalence. As for recent groundwater, these specifications are not linked to geology or depth but to the groundwater isolation in the deeper compartment as compared to surface opened groundwater. Although the occurrence of Gallionellaceae is restricted to narrow geochemical conditions (low O_2_ and high Fe concentrations; Melton et al., 2014), their occurrence in three different sites indicates that such conditions may be widespread in hard-rock aquifers. Gallionellaceae have been shown to occupy redoxicline habitats between reducing groundwater Fe-rich and oxygenated context. This is the case for groundwater inputs to freshwater systems or underground experiments (James and Ferris, 2004; Anderson et al., 2006; Sahl et al., 2008; Duckworth et al., 2009; Roden et al., 2012; Fleming et al., 2014; Reis et al., 2014; Ionescu et al., 2015). Microbial investigation of deep granitic groundwater ecosystems have shown the dominance of sulfur or iron reducing bacteria (Jain et al., 1997; Pedersen, 1997; Nyyssönen et al., 2012). We here document the occurrence of Gallionellaceae-dominated ecosystems in aquifers over a depth range of 50–250 m. This ecosystem is related to the occurrence of mixing with recent groundwater which is expected to decrease with depth. It may be expected that further down, the ecosystem observed may shift to more reducing conditions with adapted micro-organisms. We interpret the observed ecosystem as a transition zone which extends over several hundred of meters below the recent topography-dominated upper section of hard rock aquifers.

The Ploemeur site also demonstrates the effect of perturbations on groundwater bodies due to pumping-induced mixing processes, with a local mixing of recent and old groundwater strongly impacting the community structure. As mentioned earlier, near the P-R2 well more than 2500 m^3^ of water are pumped daily causing mixing of recent and old groundwater (Le Borgne et al., 2006). Although P-R1 and P-R2 groundwater chemistry are similar, P-R2 groundwater shows a taxonomic composition distinct from even nearby recent groundwater samples (P-R1, **Figure 4A**). This mixing is associated with the absence of taxa that dominate the other recent groundwater samples, such as Firmicutes. This indicates a complex relationship between nutrient fluxes disturbance induced by human activity, and the microbial community structure. It suggests that the transition from “recent” to “old” is more of a threshold than a gradient in regards to microbial communities. Identifying the point of transition from recent to old groundwater remains to be investigated, and calls for more spatially intensive sampling in a single aquifer.

## Conclusion

In order to explore the relationships between microbial community structure and groundwater position along hydrogeologic loops, we investigated the composition of the active microbial communities in three hard-rock aquifers with contrasting geology and hydrologic conditions. The data and analyses presented here reveal highly diverse bacterial community compositions in hard-rock aquifers. Microbial community structure in hard-rock aquifers seems to be intrinsically related to groundwater residence time or to the length of hydrogeologic loop, which determines the fluxes of nutrients, energy, and the delivery of microorganisms originating from the surface. Considering groundwater residence time and working at a regional scale revealed consistent patterns in microbial community structure, despite the multi-scale spatial heterogeneity inherent to hydrogeologic systems. Microbial communities in recent groundwater might be constrained by rapid and persistent transfers of nutrients and microorganisms occurring from surface, independently of aquifer lithology. In old groundwater with large residence time, isolation leads to a profound shift of microbial community composition. Our results also indicate that a constant mixing of a small percentage of recent groundwater with old groundwater, favors iron oxidation by the Gallionellaceae. Additional sequencing of metagenomes and metatranscriptomes will allow to understand the significance of autotrophic iron oxidizers in groundwater of hard-rock aquifers, and to decipher the relationships between iron oxidation and other biogeochemical cycles.

## Conflict of Interest Statement

The authors declare that the research was conducted in the absence of any commercial or financial relationships that could be construed as a potential conflict of interest.
