# Supplementary material for: Groundwater Isolation Governs Chemistry and Microbial Community Structure along Hydrologic Flowpaths
Source: Front Microbiol. 2015 Dec 22;6:1457. doi: 10.3389/fmicb.2015.01457 (PMC4686674; doi:10.3389/fmicb.2015.01457)
Supplement: Supplementary file 1 [file Presentation_1.PDF]

Supplementary Material

Groundwater isolation governs chemistry and microbial community structure along regional hydrologic flowpaths

Sarah Ben Maamar, Luc Aquilina, Achim Quaiser, Hélène Pauwels, Sophie Michon-Coudouel, Virginie Vergnaud-Ayraud, Thierry Labasque, Clément Roques, Benjamin W. Abbott, Alexis Dufresne*.

*** Correspondence:** Alexis Dufresne: Alexis.Dufresne@univ-rennes1.fr

1 Supplementary Figures and Tables

1.1 Supplementary Figures

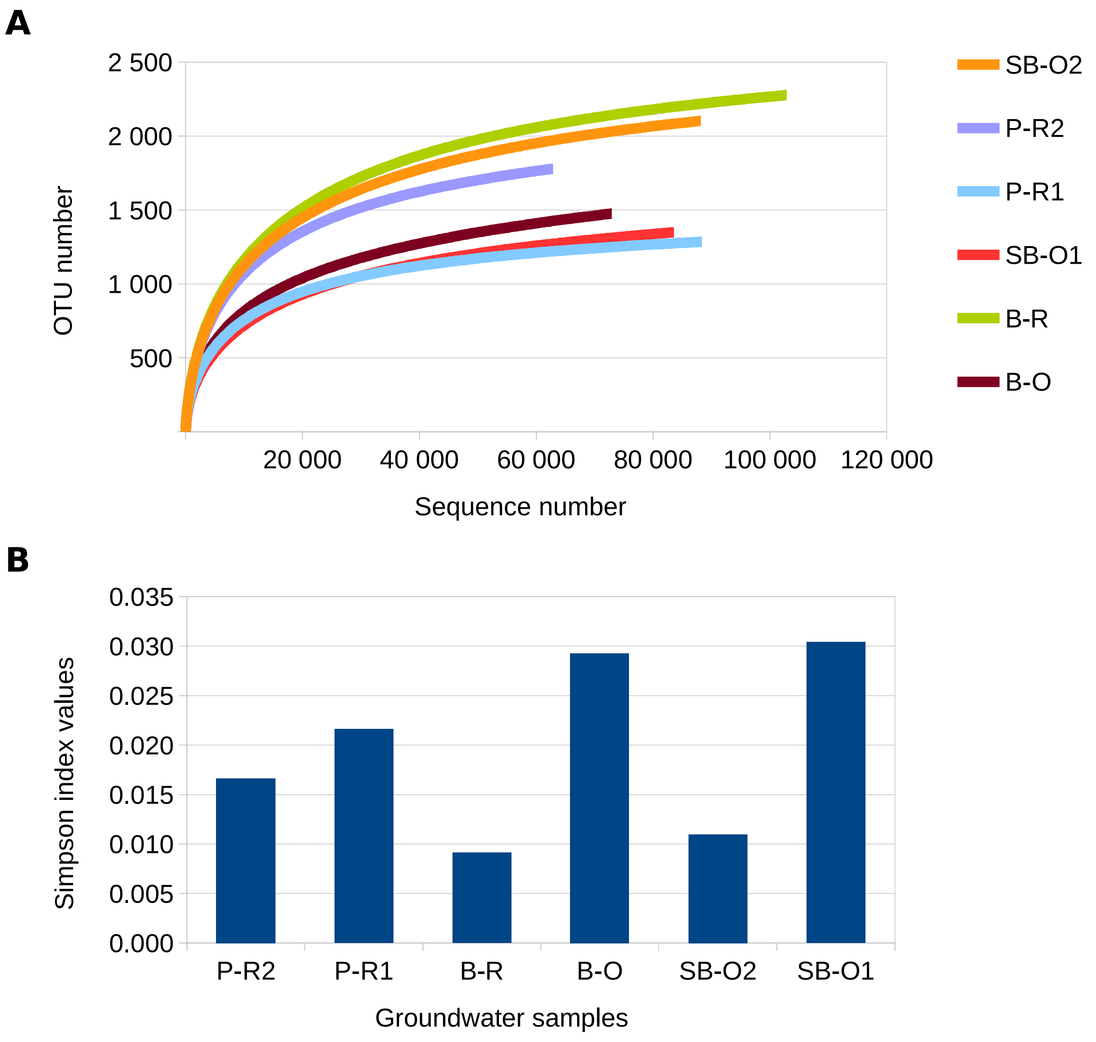

Supplementary Figure 1. Richness and species dominance of sampled groundwaters. (**A**) Rarefaction curves were used to compare species richness between the different sites. (**B**) Species dominance revealed through Simpson index values of the different groundwater samples.

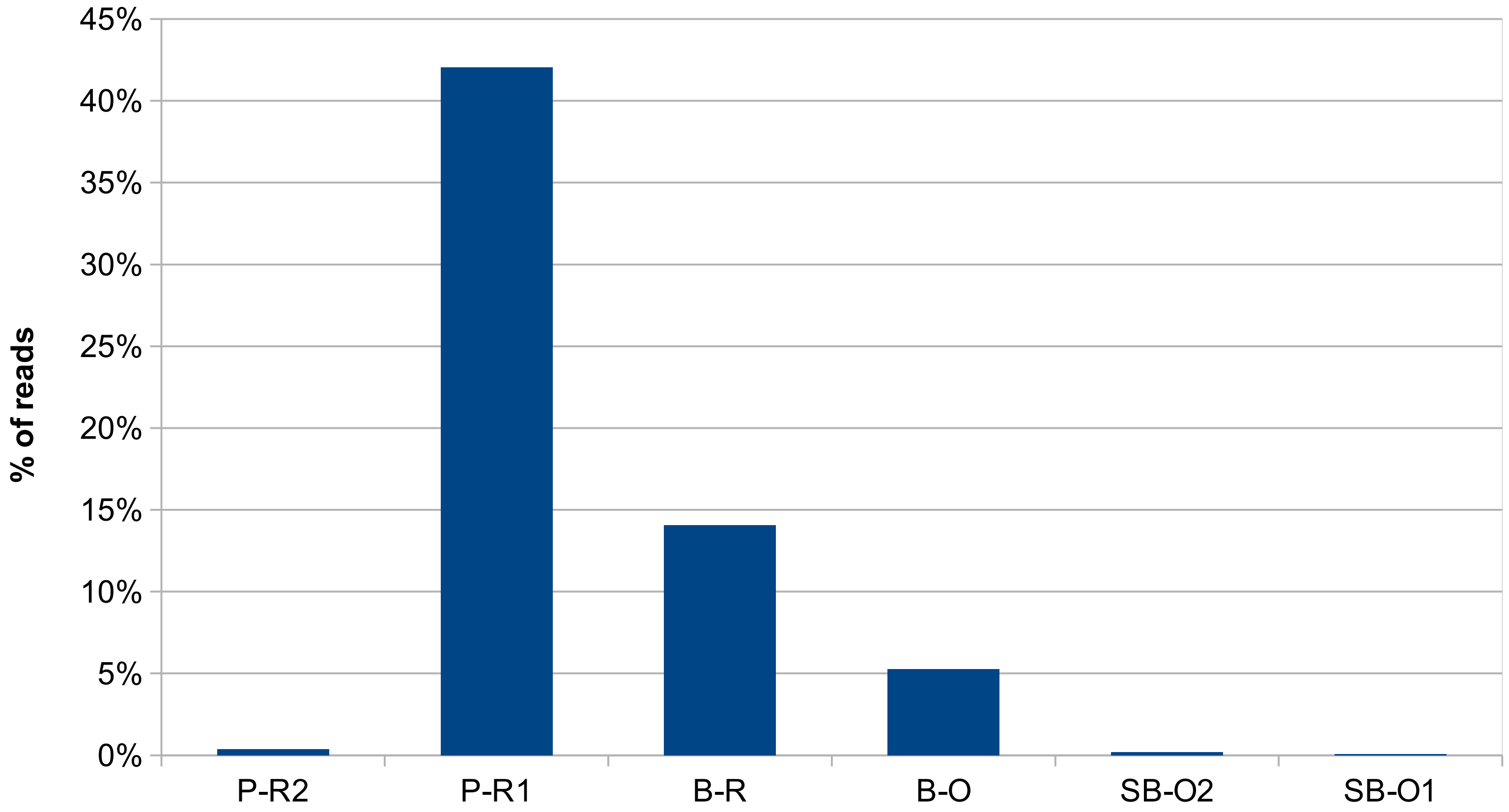

Supplementary Figure 2.

Mean percentage of sequences assigned to the *Clostridium* genus (Firmicutes).

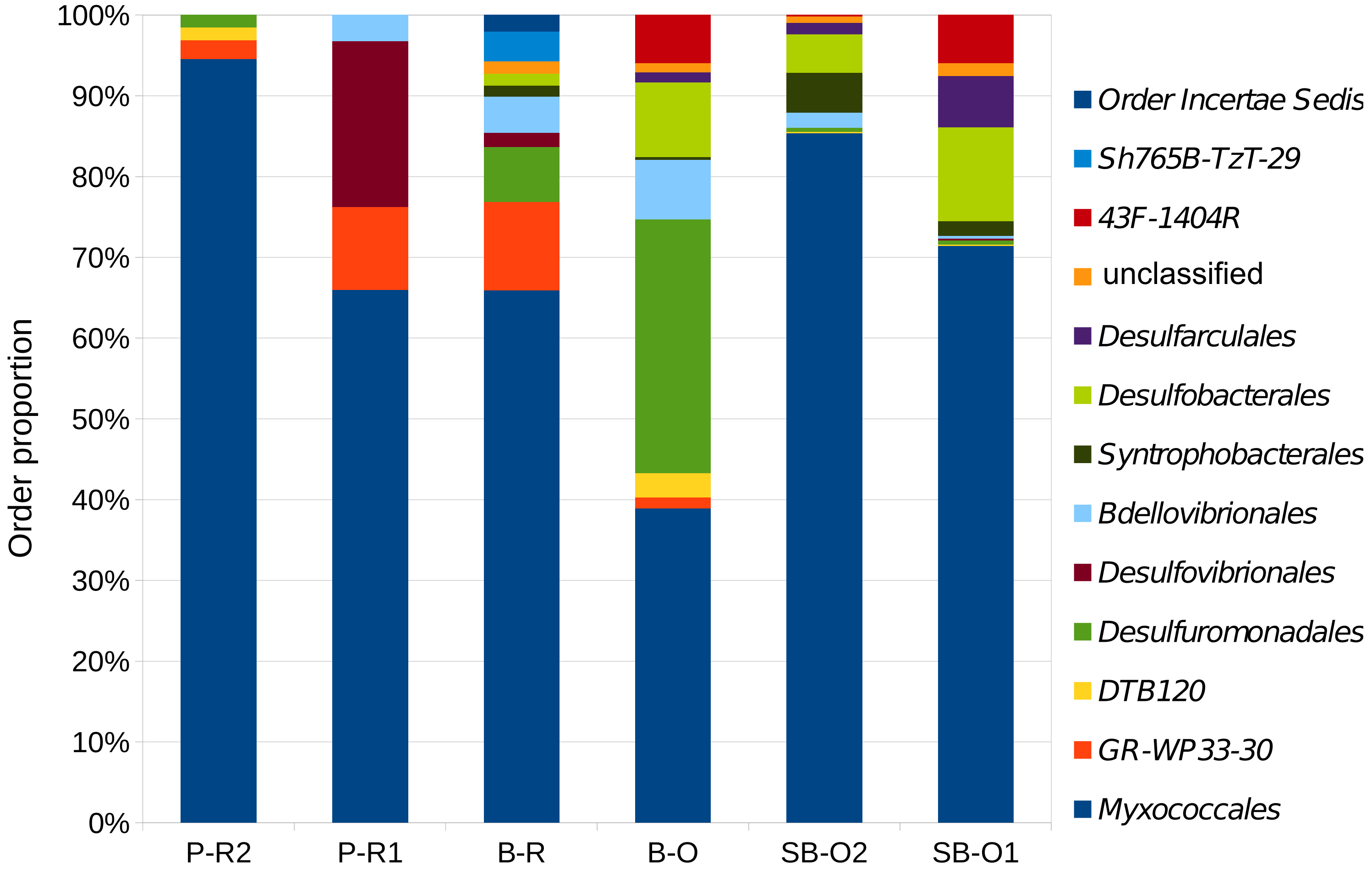

Supplementary Figure 3. Percentages of *Deltaproteobacteria* orders in the different groundwater samples. All percentages are given relatively to the total number of sequences of *Deltaproteobacteria* in each sample.

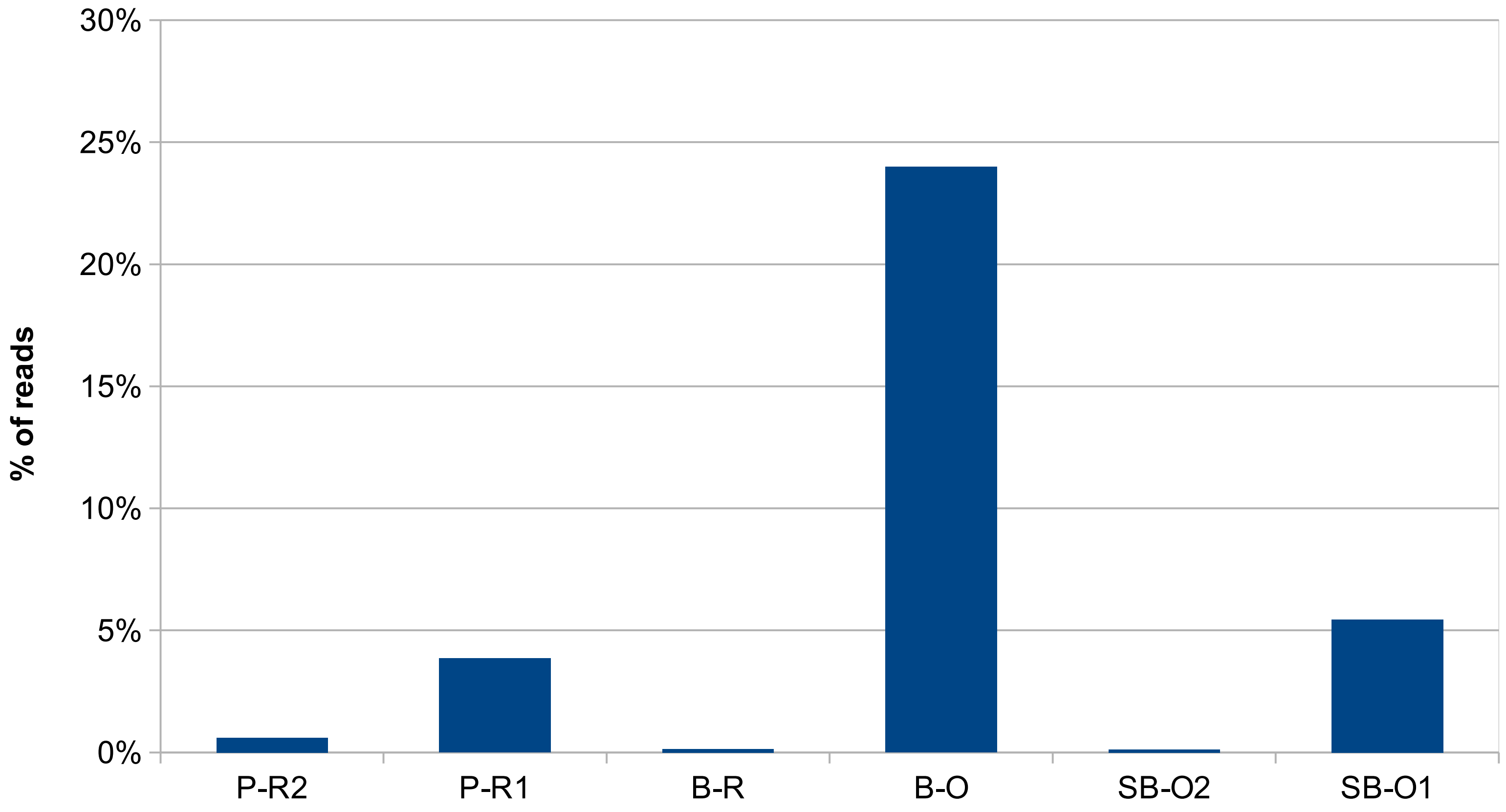

Supplementary Figure 4. Mean percentage of sequences assigned to the *Anaeromyxobacter* genus within the Myxococalles *(*Deltaproteobacteria).

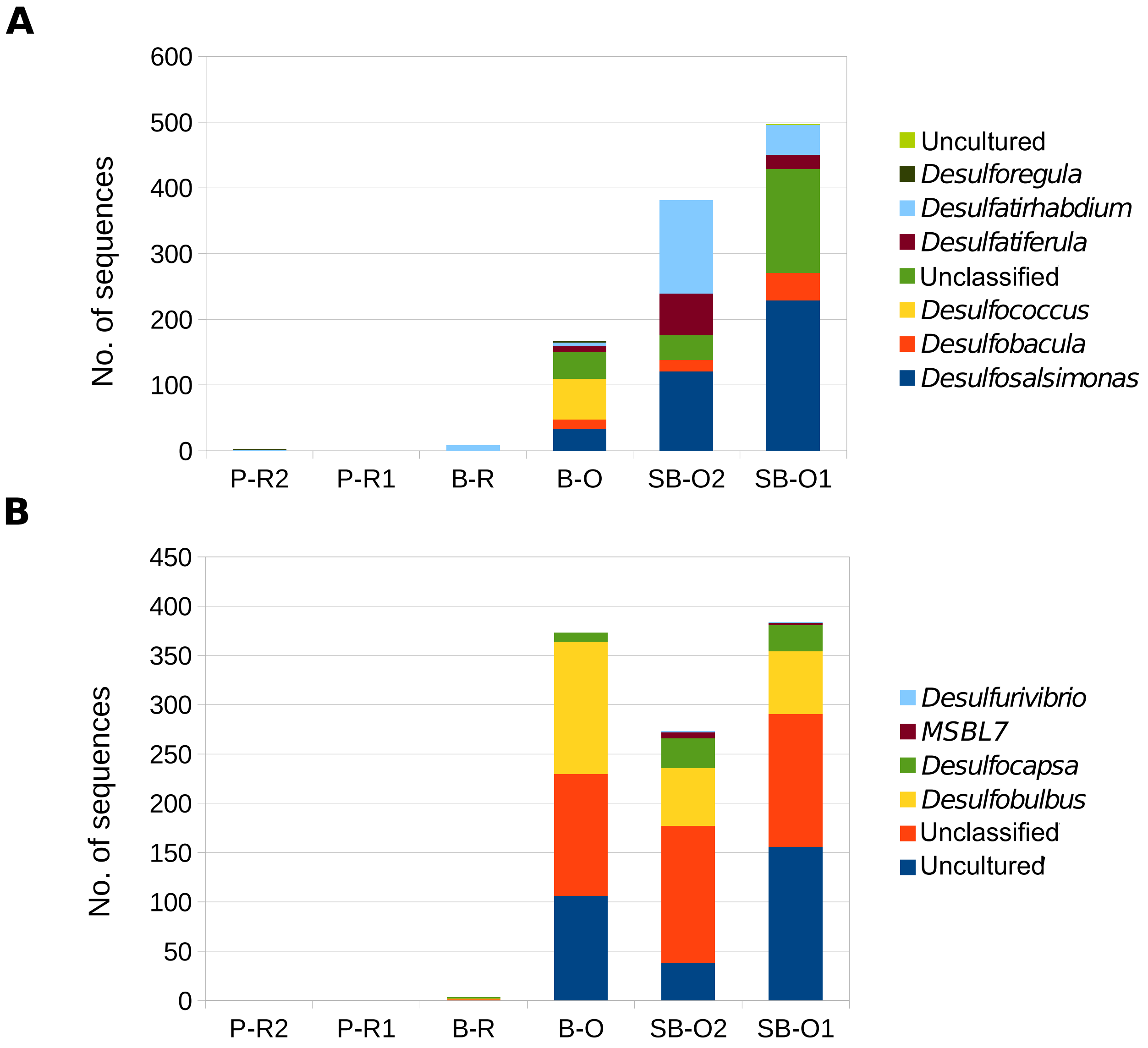

Supplementary Figure 5. Number of sequences assigned to the genera belonging to **A.** the *Desulfobacteraceae* family, and **B.** the *Desulfobulbaceae* family.

1.2 Supplementary Tables

Supplementary Table 1. Correlations between hydrogeochemical parameters characterizing the different analyzed groundwater samples. R^2 values were calculated using the cor function of the R stats package. Only parameters showing R^2 inferior to 0.80 were included in the PCA analysis. T: Temperature in °C ; Cond: Conductivity in µS/cm.

	T°C	Cl	NO_3	SO_4	Na	Mg	K	Ca	Mn	Fe	Cu	Zn	pH	Eh	O_2	Cond	Al	Si	Co	Br	Cr	Ni	V	Sr	Pb	U	Cd	Ba	Rb	Ca/Na	Br/Cl
**T°C**	1																														
**Cl**	-0,06	1																													
NO_3	-0,03	-0,25	1																												
SO_4	-0,02	0,97	-0,40	1																											
**Na**	-0,05	1,00	-0,27	0,97	1																										
**Mg**	0,26	0,78	-0,34	0,85	0,78	1																									
**K**	0,10	0,92	-0,09	0,89	0,92	0,76	1																								
**Ca**	0,01	0,93	-0,32	0,95	0,93	0,92	0,83	1																							
**Mn**	0,00	-0,19	-0,19	-0,14	-0,18	-0,16	-0,18	-0,20	1																						
**Fe**	-0,16	-0,16	-0,57	-0,14	-0,15	-0,31	-0,37	-0,22	0,25	1																					
**Cu**	-0,04	0,00	0,26	-0,05	-0,01	-0,05	0,10	-0,06	-0,11	-0,25	1																				
**Zn**	0,23	-0,18	0,18	-0,13	-0,17	0,03	0,09	-0,14	-0,10	-0,49	0,26	1																			
**pH**	-0,05	0,45	-0,47	0,50	0,45	0,36	0,32	0,42	0,01	0,42	-0,07	-0,16	1																		
**Eh**	0,17	-0,32	0,57	-0,36	-0,32	-0,17	-0,06	-0,34	0,05	-0,60	0,24	0,53	-0,60	1																	
O_2	0,12	-0,24	0,84	-0,37	-0,26	-0,27	-0,05	-0,32	-0,09	-0,59	0,20	0,35	-0,46	0,66	1																
**Cond**	0,00	0,95	-0,26	0,94	0,94	0,77	0,85	0,91	-0,18	-0,18	-0,03	-0,16	0,43	-0,37	-0,29	1															
**Al**	0,00	-0,09	0,24	-0,11	-0,10	0,08	-0,16	0,05	-0,14	-0,17	-0,06	-0,21	-0,22	0,11	0,17	-0,08	1														
**Si**	0,22	-0,54	-0,50	-0,44	-0,51	-0,41	-0,51	-0,54	0,35	0,66	-0,14	0,03	0,10	-0,19	-0,42	-0,52	-0,34	1													
**Co**	-0,41	-0,10	-0,20	0,03	-0,10	-0,06	-0,13	-0,02	0,04	-0,13	0,03	0,23	0,01	-0,01	-0,18	-0,09	-0,07	0,01	1												
**Br**	-0,05	1,00	-0,25	0,97	1,00	0,78	0,93	0,92	-0,18	-0,18	0,01	-0,14	0,45	-0,29	-0,24	0,95	-0,11	-0,52	-0,10	1											
**Cr**	-0,03	0,68	-0,09	0,65	0,70	0,52	0,69	0,61	-0,16	-0,22	0,03	0,00	0,22	-0,17	0,00	0,60	-0,10	-0,40	-0,05	0,68	1										
**Ni**	0,47	0,11	0,18	0,14	0,11	0,42	0,24	0,27	-0,14	-0,57	0,03	0,34	-0,25	0,21	0,16	0,24	0,07	-0,24	-0,27	0,11	0,09	1									
**V**	0,02	0,68	-0,27	0,71	0,71	0,60	0,72	0,67	-0,08	-0,21	-0,02	0,05	0,24	-0,16	-0,18	0,61	-0,14	-0,28	0,02	0,70	0,86	0,19	1								
**Sr**	-0,08	0,99	-0,28	0,97	1,00	0,77	0,91	0,93	-0,18	-0,13	-0,01	-0,17	0,46	-0,34	-0,28	0,93	-0,09	-0,51	-0,08	0,99	0,69	0,10	0,72	1							
**Pb**	-0,14	-0,08	0,44	-0,11	-0,09	-0,06	0,06	-0,10	0,00	-0,39	0,20	0,35	-0,15	0,38	0,42	-0,12	-0,01	-0,29	0,07	-0,07	0,07	0,14	0,04	-0,09	1						
**U**	0,63	0,05	-0,21	0,19	0,07	0,52	0,28	0,20	0,01	-0,38	0,09	0,59	0,05	0,30	-0,04	0,07	-0,14	0,17	-0,01	0,08	0,11	0,55	0,26	0,05	0,07	1					
**Cd**	0,10	0,02	-0,10	0,09	0,02	0,30	0,11	0,13	0,04	-0,17	0,03	0,24	0,08	0,15	0,03	0,02	-0,06	0,08	-0,06	0,04	0,04	0,27	0,12	0,02	0,15	0,51	1				
**Ba**	-0,37	-0,36	-0,36	-0,33	-0,36	-0,60	-0,50	-0,47	0,25	0,79	-0,14	-0,28	0,23	-0,41	-0,41	-0,34	-0,26	0,61	0,29	-0,36	-0,36	-0,72	-0,37	-0,34	-0,22	-0,50	-0,28	1			
**Rb**	0,02	0,79	-0,27	0,82	0,81	0,65	0,88	0,70	-0,02	-0,27	0,02	0,13	0,35	-0,12	-0,18	0,72	-0,30	-0,29	0,15	0,81	0,64	0,09	0,73	0,80	0,12	0,30	0,13	-0,26	1		
**Ca/Na**	-0,29	-0,54	-0,08	-0,47	-0,55	-0,28	-0,74	-0,31	0,03	0,25	-0,18	-0,24	-0,18	-0,13	-0,14	-0,53	0,42	0,15	0,36	-0,57	-0,43	-0,21	-0,42	-0,51	-0,08	-0,28	-0,05	0,26	-0,66	1	
**Br/Cl**	0,08	-0,07	0,10	-0,04	-0,06	-0,09	0,15	-0,17	0,18	-0,06	0,17	0,38	0,21	0,28	0,14	-0,08	-0,29	0,14	0,18	-0,02	0,02	-0,20	0,05	-0,08	0,19	0,26	0,15	0,20	0,28	-0,39	1

Supplementary Table 2. Hydrochemical and hydrologic characteristics of the groundwater samples. All chemical parameters are given in mg/L unless otherwise specified. Uncertainties are: 0.1 mg/L for O_2 ; 0.01 mg/L for cations; 0.1 mg/L for anions ; 3 years for Residence Time ; 0.1 unit for pH ; 0.1 °C for temperature ; 1 mV for Eh ; 0.1 µS/cm for conductivity ; 0.01 mg/L for Organic Carbon and Inorganic Carbon.

Well	Site	Sampling depth (m)	R.D. (m)	Compartment	Mean R.T. (years)	pH	Eh (mV)	Cond (µS/cm)	Cl	SO_4	NO_3	O_2	IC	TOC	Fe	Na
**P-R2**	**Ploemeur**	68	40	Fractured	16	5.5	370	346	57.0	9.4	47.1	3.2	0	0.41	0	36.6
**P-R1**		65	35	Fractured	16	5.5	356	307	45.3	11.3	47.4	5.9	0.03	0.24	0	32.7
**P-O**		90	55	Fractured	46	6.	174	616	38.6	41.5	1.0	1.6	21.3	0.00	0	71.5
**B-R**	**Betton**	13	1	Weathered	16	5.9	259	390	42.4	25.1	52.9	6.1	8.8	0.42	0.1	23.7
**B-O**		70	48	Fractured	48	7.4	35	3230	737.1	291.1	0.0	0.3	30.7	0.00	1.3	540.1
**SB-O2**	**Saint-Brice-En-Coglès**	216	68	Fractured	55	6.3	12	227	21.5	19.3	3.9	0.3	18.4	0.56	5.2	16.7
**SB-O1**		80	36	Fractured	62	6.4	6.4	237	22.6	21.5	5.8	0.2	19.2	0.49	5.9	18.4
**SB-O**		15	5	Fractured	53	6.2	40	221	21.5	19.4	13.9	0.4	17.3	0.65	1.5	18.0

R.D. = Relative Depth, corresponding to sampling depth relatively to the weathered and fractured zone transition.
R.T. = groundwater Residence Time.
O_2 corresponds to dissolved O_2.
Fe corresponds to total dissolved iron .
IC = Inorganic Carbon
TOC = Total Organic Carbon
